# Reference ranges for biventricular volumes and ejection fraction and for left ventricular mass in adult thalassemia intermedia patients without myocardial iron overload

**DOI:** 10.1186/1532-429X-15-S1-P102

**Published:** 2013-01-30

**Authors:** Antonella Meloni, Petra Keilberg, Gaetano Giuffrida, Angelo Peluso, Cristina Salvatori, Gennaro Restaino, Gianluca Valeri, Vincenzo Positano, Daniele De Marchi, Letizia Gulino, Massimo Lombardi, Alessia Pepe

**Affiliations:** 1 CMR Unit, Fondazione G.Monasterio CNR-Regione Toscana and Institute of Clinical Physiology, Pisa, Italy; 2 Divis. Clinicizzata di Ematologia, Ospedale "Ferrarotto", Catania, Italy; 3 Microcitemia, Presidio Ospedaliero Centrale, Taranto, Italy; 4 Fondazione G.Monasterio CNR-Regione Toscana, Pisa, Italy; 5 Istituto di Radiologia, Centro di Ricerca e Formazione ad Alta Tecnologia nelle Scienze Biomediche "Giovanni Paolo II" - Università Cattolica del Sacro Cuore, Campobasso, Italy; 6 Istituto di Radiologia, Azienda Ospedaliero-Universitaria Ospedali Riuniti "Umberto I-Lancisi-Salesi", Ancona, Italy

## Background

Thalassemia intermedia (TI) patients were shown to have significantly higher cardiac output and cardiac volumes with respect to thalassemia major (TM) patients. So, to compare biventricular parameters in TI patients with established ranges from TM may be misleading.

The aim of this study was to establish the ranges for normal biventricular volumes and ejection fraction (EF) and for left ventricular (LV) mass assessed by cardiovascular magnetic resonance (CMR) in TI.

## Methods

Among the 294 TI patients with > 18 years of age enrolled in the Myocardial Iron Overload (MIOT) network who underwent CMR, we selected 68 patients with no known risk factors or history of cardiac disease, normal electrocardiogram, no myocardial iron overload (all the cardiac segments with a normal T2* value) and no myocardial fibrosis. Biventricular parameters were quantitatively evaluated in a standard way by SSFP cine images using MASS® software. LV and right ventricular (RV) end-diastolic volume (EDV), end-systolic volume (ESV) and stroke volume (SV) were normalized by body surface area (EDVI, ESVI, SVI), as well as the LV mass.

## Results

The selected patients had a mean age of 36.5 ± 9.2 and 37 were males.

Biventricular volumes indexes were significantly larger in males than in females, with the exception of the RV ESVI. The LV mass was significant higher in males while the LV and the RV EFs were not different between the sexes. No significant differences among TI regularly (N=36), sporadically (N=16) and no transfused (N=16) were found in biventricular parameters, so no division was made on the basis of the transfusional regimen.

The biventricular parameters are detailed in table 1 with differentiation for sex. Table 1 reports also the cut-off of normality defined as mean - 2 standard deviation (SD) for the volumes and the LV mass and as mean - 1 SD for the EF (considering the high cardiac output state in anemic patients).

**Table 1:** 

	Males			Females		
	**Mean ± SD**	**95% CI**	**Normal value**	**Mean ± SD**	**95% CI**	**Normal value**

***LV EDVI(ml/m^2^)***	101.5 ± 22.6	93.9 -109.0	<147	89.4 ± 15.1	83.8 - 94.9	<120

***LV ESVI(ml/m^2^)***	38.1 ± 11.2	34.4 - 41.8	<60	32.2 ± 7.9	29.3 - 35.1	<48

***LV SVI(ml/m^2^)***	63.6 ± 13.9	58.9 - 68.2	<91	57.4 ± 12.3	52.9 - 61.9	<82

***LV Mass I(g/m^2^)***	69.2 ± 12.6	64.9 - 73.4	<94	57.7 ± 10.9	53.7 - 61.8	<79

***LV EF(%)***	62.6 ± 5.6	60.7 - 64.4	>57	63.8 ± 5.9	61.6 - 66.0	>57.9

***RV EDVI(ml/m^2^)***	94.7 ± 19.8	88.1 - 101.3	<134	83.9 ± 16.1	77.3 - 90.6	<116

***RV ESVI(ml/m^2^)***	33.6 ± 8.1	30.9 - 36.3	<50	30.5 ± 9.3	27.2 - 33.9	<49

***RV SVI(ml/m^2^)***	61.1 ± 14.8	56.2 - 66.1	< 91	52.9 ± 13.6	47.9 - 57.9	<80

***RV EF(%)***	63.9 ± 5.7	62.1 - 65.9	>58.2	63.2 ± 7.9	60.3 - 66.1	>55.3

## Conclusions

Reference ranges for biventricular volumes and function specific to adult TI patients were defined. These new reference ranges are important for avoiding a misdiagnosis of cardiomyopathy in TI patients.

## Funding

The MIOT project receives "no-profit support" from industrial sponsorships (Chiesi and Apotex). This study was also supported by: "Ministero della Salute, fondi ex art. 12 D.Lgs. 502/92 e s.m.i., ricerca sanitaria finalizzata anno 2006" e "Fondazione L. Giambrone".

